# The Hospitals Association

**Published:** 1888-01-28

**Authors:** 


### The Hospitals Association.

THE following statement, signed by the Secretary on behalf of the President and Council of The Hospitals Association, was ordered to be issued at a meeting of the Council held last week :—

"The Council of The Hospitals Association see with regret that an attempt is being made to misconstrue their motives in furthering the work of the society, which was formed some years since, mainly to diffuse a sound knowledge of hospital economy, and to strengthen and widen the public interest in and support of hospitals. Amongst other objects the Council had in view, not the least important was an endeavour to promote by every means in its power the comfort and well-being of hospital nurses. Feeling strongly that something was required to secure for nurses generally home comforts in sickness and in old age, it saw the necessity which existed for the establishment of a provident fund to which nurses of all ages might have the power of contributing, and afterwards reap the benefit. It was also considered of essential importance that a scheme of registration for trained and certificated nurses should be inaugurated, and for this purpose the Council appointed a sectional committee of ladies engaged in the training and superintendence of nurses in London and provincial hospitals to discuss the subject from a practical point of view and to tabulate their suggestions. The recommendations of this committee, slightly modified by the Council, resulted in a set of rules, which were unanimously adopted on May 17th last, at a joint meeting of the Council and sectional committee, at which the latter formed the majority. From some ambiguity, however, in the terms employed to define the period of probation recommended to be pursued by all nurses in training establishments, exception has since been taken to the action of the Council, and some of the ladies engaged on the sectional committee have thought proper to withdraw altogether from the Association. But in order that the question may be reconsidered and discussed in all its bearings, and with fuller information at command, a new and representative joint committee of ladies and gentlemen engaged in hospital and nurse-training work has been formed with instructions to confer with the representatives of the governors and heads of the nursing departments in the leading hospitals in London and the provinces, with the view of organising a register for nurses, which shall be acceptable to the greater number of these establishments.

"Thus the aims and work of the Association (which includes among its members clergymen, medical men, presidents, treasurers, governors, superintendents, secretaries and matrons of hospitals, together with many other persons interested in hospital management) were intended to be, and are, purely philanthropic. And it is gratifying to the Council to be able to state that many interesting and important questions have engaged their attention in addition to those beneficial objects to which attention has here been particularly directed, and which they have reason to believe are in course of realization."

---

FATS AS TONICS.—Fats, especially those that are easy of digestion, like cod-liver oil and sweet cream, are essential to the well-being of the nervous system. The peculiar substance —neurine—found in all nervous structures contains fat as an essential constituent. It is remarkable that most nervous individuals have a strong aversion to fats as articles of diet. This is extremely unfortunate, for the omission of fats and oils from the diet tends not only to continue the nervousness, but to increase the irritability and weakness. Cod-liver oil is a most valuable medicine in such cases, because it is already partly digested by admixture with the bile secreted in the liver of the fish, and thus rendered still more easy of absorption. The labour of digestion is thus partly taken away from the task to be performed by the invalid.

